# It Takes More Than a Good Camera: Which Factors Contribute to Differences Between Face-to-Face Interviews and Videoconference Interviews Regarding Performance Ratings and Interviewee Perceptions?

**DOI:** 10.1007/s10869-020-09714-3

**Published:** 2020-09-10

**Authors:** Johannes M. Basch, Klaus G. Melchers, Anja Kurz, Maya Krieger, Linda Miller

**Affiliations:** Institut für Psychologie und Pädagogik, Universität Ulm, Albert-Einstein-Allee 41, D-89069 Ulm, Germany

**Keywords:** Technology-mediated interviews, Impression management, Social presence, Privacy concerns, Job interviews, Applicant reactions

## Abstract

Due to technological progress, videoconference interviews have become more and more common in personnel selection. Nevertheless, even in recent studies, interviewees received lower performance ratings in videoconference interviews than in face-to-face (FTF) interviews and interviewees held more negative perceptions of these interviews. However, the reasons for these differences are unclear. Therefore, we conducted an experiment with 114 participants to compare FTF and videoconference interviews regarding interview performance and fairness perceptions and we investigated the role of social presence, eye contact, and impression management for these differences. As in other studies, ratings of interviewees’ performance were lower in the videoconference interview. Differences in perceived social presence, perceived eye contact, and impression management contributed to these effects. Furthermore, live ratings of interviewees’ performance were higher than ratings based on recordings. Additionally, videoconference interviews induced more privacy concerns but were perceived as more flexible. Organizations should take the present results into account and should not use both types of interviews in the same selection stage.

Like many other fields, personnel selection has undergone a considerable transformation due to the tremendous technological progress in recent years (Ryan et al., 2015; Stone, Deadrick, Lukaszewski, & Johnson, 2015). Nowadays, interviews are no longer only conducted face-to-face (FTF), but videoconferencing technologies and, more generally, the internet, offer many opportunities to make the recruitment process and interviews more flexible in terms of time and place (Blacksmith, Wilford, & Behrend, 2016; Chamorro-Premuzic, Winsborough, Sherman, & Hogan, 2016).

Although so-called technology-mediated interviews offer many advantages, previous studies have found that interviewees usually receive lower ratings in these interviews compared to FTF interviews (Blacksmith et al., 2016; Sears, Zhang, Wiesner, Hackett, & Yuan, 2013). These differences in the rated interview performance between interviews might eventually have negative consequences for personnel selection if different kinds of interviews are used for the same pool of applicants during the same selection stage. However, the reasons for these differences are unclear.

In addition to differences concerning interview performance ratings, previous research also revealed that applicants are more skeptical about technology-mediated interviews than about FTF interviews (Blacksmith et al., 2016). As potential reasons for the more skeptical perceptions of technology-mediated interviews, a lack of social presence (i.e., an impaired feeling of the physical awareness of one’s conversation partner) and impairments of the use of impression management (IM) tactics have been suggested. More specifically, it is assumed that interviewees in technology-mediated interviews are feeling less comfortable regarding the use of tactics to present themselves in a positive manner in the same way they would in a FTF interview (Basch, Melchers, Kegelmann, & Lieb, 2020). These negative perceptions of technology-mediated interviews can have negative consequences for organizations given that previous studies found that potential applicants may decide not to take up a position or to talk badly about the hiring organization (Hausknecht, Day, & Thomas, 2004).

To gain further insights into the causes of the lower performance ratings and more negative perceptions, the present study compared FTF and videoconference interviews regarding interview performance and fairness perceptions. Besides the mere comparison, we also took a closer look at potential reasons for the differences concerning interview performance and fairness perceptions.

## Background

### Technology-Mediated Employment Interviews

Employment interviews are the most widely used tool for personnel selection. Almost every organization uses them at some point during the selection process, often even as the only personnel selection tool (Huffcutt & Culbertson, 2011; Levashina, Hartwell, Morgeson, & Campion, 2014). Furthermore, the popularity of interviews for personnel selection is no coincidence. In addition to their ease of implementation, they are well accepted by applicants compared to other selection tools (Anderson, Salgado, & Hülsheger, 2010; Huffcutt & Culbertson, 2011) and well-designed interviews can also predict later work performance very well (Huffcutt, Conway, Roth, & Klehe, 2004; Huffcutt, Culbertson, & Weyhrauch, 2014).

Due to technological progress, alternatives to FTF interviews have been developed that offer several advantages for organizations as well as for applicants (Potosky, 2008). These alternatives include telephone interviews (e.g., Straus, Miles, & Levesque, 2001), interviews by interactive voice response (IVR) systems (e.g., Bauer, Truxillo, Paronto, Weekley, & Campion, 2004), videoconference interviews (e.g., Sears et al., 2013), and so-called asynchronous video interviews (also called digital interviews, Langer, König, & Krause, 2017, or video interviews, Toldi, 2011). In these interviews, candidates are shown pre-defined questions on the screen and their answers are recorded via webcam and microphone so that they can be evaluated by the interviewer later (e.g., Brenner, Ortner, & Fay, 2016).

Of the different ways in which interviews can be conducted, videoconference interviews are probably one of the most obvious alternatives to FTF interviews. Nowadays, most applicants are used to videoconferencing technologies such as Skype, Google Hangouts, or FaceTime, and the rapid technological advances during recent years have steadily improved the quality of connection and communication. In these systems, the connection between the call partners is established via the internet. Although the interview partners are not in the same room during videoconference interviews and although the screen does not show a complete picture of the conversation partners, the exchange of communication through the use of webcams and microphones can come relatively close to FTF interviews.

Videoconference interviews are more comparable to FTF interviews with regard to many properties in comparison to other ways in which interviews can be administered. Accordingly, they are often used during similar selection stages. Nevertheless, as is explained below, previous research revealed performance differences between videoconference versus FTF interviews as well as differences in interviewees’ perceptions of these interviews. Therefore, the present study focuses on a comparison between these two types of interviews.

### Theoretical Background of Differences Between FTF and Videoconference Interviews

Huffcutt, Van Iddekinge, and Roth’s (2011) interviewee performance model can be used as a theoretical framework that covers various aspects that affect interview performance ratings so that it is also a useful starting point for a comparison between FTF and videoconference interviews. Within the model, different factors can be distinguished on both the interviewer and the interviewee side that can influence ratings of interviewees’ performance. On the interviewee side, for example, tactics such as IM can be used to positively influence evaluations in the interview. On the interviewer side, memory limitations or different biases can influence ratings of interviewees’ performance. In addition, individual factors (such as interviewees’ general mental ability, personality, appearance), interactive factors (such as mutual sympathy of interviewers and applicants), and situational factors such as the interview medium are considered in this model.

Although conversation quality in videoconference interviews is nowadays often relatively close to FTF interviews due to high-resolution cameras and high-speed internet, there are still some aspects that might be different. In the following, we therefore take a closer look at common media communication theories to be better able to distinguish between these two kinds of interviews.

According to social presence theory (Short, Williams, & Christie, 1976), different interview media may vary with regard to the level of perceived social presence. Short et al. define social presence as “the degree of salience of the other person in the interaction and the consequent salience of the interpersonal relationships” (p. 65). Moreover, social presence represents the “mental set” (Short et al., 1976, p. 66) of an individual about a certain communication situation including the summary of the awareness of the other person with regard to gaze, para-verbal information, facial expressions, and gestures. Furthermore, Short et al. assumed that social presence suffers under the influence of telecommunication.

The assumption of social presence theory is that communication through a medium creates a sense of togetherness (Biocca, Harms, & Burgoon, 2003). However, this togetherness is not fully comparable with the feeling of the physical presence of one’s conversation partner. Furthermore, part of this togetherness refers to the feeling of reciprocal communication that also concerns non-verbal behaviors, which might be restricted in videoconference interviews because of not showing the complete image of one’s gestures and facial expressions. Accordingly, it may be that the social component of an interview suffers because of the spatial separation of interviewees and interviewers and the technological mediation (Bauer, Truxillo, Mack, & Costa, 2011; Melchers, Ingold, Wilhelmy, & Kleinmann, 2015).

Potosky’s (2008) framework of media attributes can be used as another theoretical basis to distinguish between different interview media. According to this framework, one can differentiate media by four attributes: social bandwidth, interactivity, transparency, and surveillance. First, social bandwidth refers to an aspect that is also covered by media richness theory (Daft & Lengel, 1986): the more communication paths (verbal, non-verbal, para-verbal) are used in the transmission of information by a sender, the better the information is understood by the recipient. Second, interactivity refers to the amount of interaction that is possible between the conversation partners. Next, transparency refers to the degree to which the conversation partners are aware of technological mediation. Finally, surveillance refers to the fear that a technology-mediated conversation is recorded or monitored by a third party.

If one compares FTF interviews and videoconference interviews with regard to Potosky’s (2008) attributes, social bandwidth is better in FTF interviews because videoconference programs hardly show the complete picture of the other person, which can lead to limitations in information delivery (Toldi, 2011). In addition, lag times may lead to a limitation of interactivity in videoconference interviews (Wegge, 2006). Given that FTF interviews represent a normal conversation situation (apart from their evaluative nature), there are no restrictions to be expected concerning transparency whereas microphone and camera could mitigate transparency in videoconference interviews. Furthermore, transparency might be even more affected when applicants see their own image on the screen via a picture-in-picture window (Horn & Behrend, 2017). Finally, as soon as one uses a technical medium for a conversation, one can never be sure that the conversation will not be recorded or surveilled.

To sum this up, the quality of conversation in videoconference interviews and FTF interviews has become increasingly similar due to technological progress like high-resolution cameras and faster internet connections in recent years. However, regarding social presence theory (Short et al., 1976) and Potosky’s (2008) framework of media attributes, they still differ concerning several potentially relevant aspects.

## Review of Previous Research and Development of Hypotheses

### Ratings of Interviewees’ Performance in FTF and Technology-Mediated Interviews

As mentioned above, meta-analytic evidence has found lower performance ratings of interviewees in technology-mediated interviews compared to FTF interviews (Blacksmith et al., 2016). However, the primary studies in this meta-analysis also considered other forms of technology-mediated interviews including telephone or interactive voice response interviews. Additionally, the primary studies were all conducted at least 9 years prior to the meta-analysis at a time when interviews via videoconferencing technologies were still facing problems, such as slow internet connections. However, due to technological progress, high-speed internet, and high-resolution cameras, it is possible to create a conversation quality that can come close to FTF communication. Nevertheless, more recent studies that used up-to-date videoconference software and faster internet connections (Melchers, Petrig, & Sauer, 2016; Sears et al., 2013) still found lower performance ratings of interviewees in videoconference interviews.

As already noted above, these differences might be due to impairments of interviewees’ performance because of impression management, social presence, or eye contact. These factors will be considered in more detail below where we offer specific hypotheses related to each of them. However, it is also possible that interviewers evaluate interviewees more negatively because of technological barriers. In line with this, Van Iddekinge, Raymark, Roth, and Payne (2006) found that interview performance ratings were lower when they were made on the basis of video recordings than when they were made on the basis of own observations during the actual interview. In any case, however, and in line with previous research, we offer the following main hypothesis:

- **Hypothesis 1**. Interviewees receive lower performance ratings in videoconference interviews compared to FTF interviews.

### Interviewee Impression Management

Blacksmith et al. (2016) suggested that one reason for the lower ratings in technology-mediated interviews is that interviewees are restricted in their use of IM tactics (see Chapman & Rowe, 2002, for a similar suggestion). Usually, interviewees can use verbal IM tactics such as emphasizing potential strengths or playing down potential weaknesses or failures or non-verbal IM tactics such as making eye contact, smiling, nodding, and maintaining a specific body posture (Frauendorfer & Schmid Mast, 2015). Previous research repeatedly found that the use of such IM behaviors correlates with better interview performance ratings (Barrick, Shaffer, & DeGrassi, 2009; Burnett & Motowidlo, 1998; Levashina et al., 2014). However, in technology-mediated interviews, the use of the different IM tactics might be impaired. For example, lags concerning the internet connection and associated impairments of the conversation flow might lead to such an impairment. Similarly, delayed, weakened, or missing feedback from the interviewer, to which non-verbal behavior is usually adapted, or impaired possibilities for identifying facial cues for emotional interpretation (Fullwood & Finn, 2010) might impede interviewees’ use of IM tactics.

Although, as already mentioned, the transmission quality has improved in recent years due to technological progress, it can still be assumed that interviewees feel less comfortable using IM in technology-mediated interviews. Even if high-class cameras show a high-resolution image of the interview partner, minimal time delays can give the feeling that self-promotional statements have not been received as they should have been. Furthermore, the picture of the interviewer contains only a part of the complete picture that can be seen in a FTF interview, which impairs non-verbal IM. In line with this, results from a recent survey by Basch et al. (2020) concerning potential applicants’ views of technology-mediated interviews revealed lower perceptions of being able to use IM in technology-mediated interviews.

Based on these conceptual arguments and given that IM has turned out to be correlated with interviewer ratings in FTF interviews (Barrick et al., 2009; Levashina et al., 2014), we suspect differences of the use of IM as one of the reasons for the lower performance ratings in videoconference interviews. Accordingly, we predict the following mediation effect:

- **Hypothesis 2a**. Interviewees use lower levels of IM in videoconference interviews than in FTF interviews, which in turn leads to lower performance in videoconference interviews.

One of the already-mentioned non-verbal IM tactics is seeking eye contact, which can be defined as fixing the eyes of the communication partner (Bohannon, Herbert, Pelz, & Rantanen, 2013). Eye contact is used in conversations to obtain information and feedback (Forbes & Jackson, 1980). Furthermore, research has shown that building eye contact in recruitment interviews goes along with better ratings of interviewees’ performance (Burnett & Motowidlo, 1998; Imada & Hakel, 1977; Kleinke, 1986).

In videoconference interviews, the possibility of making eye contact is also impeded. This is partly because eye contact in videoconference interviews can only be made by looking into the camera (i.e., the gaze has to be directed away from the picture of the conversation partner) and not as usual in FTF communication by fixing the eyes of the conversation partner (Bohannon et al., 2013; McColl & Michelotti, 2019). Furthermore, in a recent study by McColl and Michelotti (2019), interviewers reported that poor eye contact affected their perception of candidates’ sincerity. In addition, the potentially poorer resolution of videoconference systems may hinder candidates from establishing eye contact (Chapman & Rowe, 2002). Based on this, we predict:

- **Hypothesis 2b**. Interviewees will perceive a lower quality of eye contact in videoconference interviews compared to FTF interviews.

If one combines the previous two hypotheses, then a serial mediation effect is predicted. Specifically, the use of videoconference interviews should affect interview performance by first leading to impaired quality of eye contact and this impaired quality of eye contact should then lead to impaired use of IM tactics. Finally, the impaired use of IM tactics should lead to lower interview ratings. Therefore, we additionally predict:

- **Hypothesis 2c**. The relationship between interview medium and interview performance ratings is serially mediated by perceived quality of eye contact and IM so that interviewees will report lower perceived quality of eye contact in videoconference interviews, which will first impair their use of IM tactics and the impaired use of IM tactics will ultimately lead to impairments of their interview performance.

### Social Presence

Blacksmith et al. (2016) suggested that social interaction in technology-mediated selection interviews is impaired because social cues are not as rich as in FTF interviews. Similarly, Melchers et al. (2016) proposed that the differences in performance ratings between different interview media that they observed in their study cannot be attributed to differences in the richness of transmitted cues, but rather to differences in perceived social presence. However, previous studies did not use any measures of social presence to evaluate the viability of these suggestions.

According to the propositions by Short et al. (1976) mentioned above, social presence is derived from the extent of the feeling of the presence of the conversation partner, which is in turn a summary of the perception of facial expressions, gestures, and also the general feeling of mutual presence. Accordingly, social presence in technology-mediated forms of communication is limited because of the physical absence of the interaction partner, the screen size, and the restrictions of the image captured by the camera. Therefore, taking the social presence theory (Short et al., 1976) and some of Potosky’s (2008) media attributes into account, there might be a lack of social presence in videoconference interviews due to spatial and technological separation of the interview partners. Therefore, we predict:

- **Hypothesis 3a**. Perceived social presence is higher in FTF interviews compared to videoconference interviews.

Furthermore, it has already been found in different contexts that a higher level of social presence goes hand in hand with better performance. For example, a greater degree of perceived social presence is associated with better collaborative learning (So & Brush, 2008). However, we are not aware of any research on the relationship between perceived social presence and performance ratings in the interview context. Nevertheless, Huffcutt et al. (2011) already suggested that interview design factors like the interview medium might influence interview performance. According to social presence theory (Short et al., 1976), communication is better when perceived social presence is higher. Therefore, conducting an interview via videoconference, which suffers from a lack of social presence, negatively influences the effectiveness of the communication between interviewer and interviewee and ultimately interview performance. Thus, we assume the perceived social presence is a mediating factor that contributes to the lower performance ratings in videoconference interviews predicted in Hypothesis 1. Therefore, we predict:

- **Hypothesis 3b**. The relationship between interview medium and interview performance ratings is mediated by perceived social presence.

In a recent survey, Basch et al. (2020) found that at least the relationship between interview medium and fairness perceptions of technology-mediated interviews are serially mediated by assumed social presence and IM. Given that interviews depend on socio-emotional interactions (Melchers et al., 2015) in which interviewees try to present themselves in a positive manner to influence interviewers, IM also depends on perceived social presence. Therefore, the serial mediation found by Basch et al. (2020) may not only hold for fairness perceptions as the dependent variable, but also for interview performance ratings. Accordingly, we predict:

- **Hypothesis 4**. The relationship between interview medium and interview performance ratings is serially mediated by perceived social presence and IM.

### Interviewees’ Perceptions of Different Interviews

The meta-analysis by Blacksmith et al. (2016) also revealed that interviewees perceive technology-mediated interviews more negatively than FTF interviews. However, this meta-analysis has mainly included studies related to telephone (e.g., Chapman, Uggerslev, & Webster, 2003) or interactive voice response interviews (e.g., Bauer et al., 2004) – or to videoconference interviews that were conducted with older videoconference technologies from the past decades (e.g., Chapman & Rowe, 2002). Therefore, it is unclear to which degree these results hold true nowadays.

In search of possible antecedents of interviewee perception differences, one might refer to Gilliland’s (1993) fairness model of applicant reactions. According to this model, fairness perceptions of a selection procedure are related to different justice rules. Furthermore, these fairness perceptions can influence important outcomes like perceived organizational attractiveness or applicants’ behavioral intentions (Hausknecht et al., 2004) and also their actual job offer acceptance (Harold, Holtz, Griepentrog, Brewer, & Marsh, 2016).

In general, interviews meet many of the justice rules that are mentioned in Gilliland’s (1993) model, like allowing two-way communication or the opportunity for interviewees to show their qualifications, experiences, and skills. However, given that communication changes through the use of technology, this also means that these rules are not fulfilled to the same degree in technology-mediated interviews (Bauer et al., 2011).

To the best of our knowledge, there are only two newer studies that compared perceptions of FTF and videoconference interviews using current videoconferencing technologies (Melchers et al., 2016; Sears et al., 2013). Nevertheless, these studies still found that interviewees report more negative perceptions of videoconference interviews compared to FTF interviews. In addition, with regard to Gilliland’s (1993) justice model, Sears et al. also found that interviewees rated videoconference interviews not only lower regarding the possibility to present themselves, but rated them as less job-relevant and less face-valid. These results were paralleled in the survey by Basch et al. (2020), which compared perceptions of FTF, videoconference, and asynchronous video interviews. In comparison to FTF interviews, fairness perceptions of videoconference interviews were lower and the difference was even stronger concerning the comparison of FTF versus asynchronous video interviews.

Regarding the theories presented earlier, it seems likely that interviewees’ perceptions are affected by restrictions of social presence and of some of the media attributes by Potosky (2008). According to social presence theory (Short et al., 1976), technology-mediation might be seen as a kind of barrier that adversely affects communication and therefore also the perception of the interview. And according to Potosky’s framework, some media attributes are also negatively affected in videoconference interviews. Social bandwidth, for example, is impaired because no complete picture of the interviewer and the interviewee is shown (Toldi, 2011). Furthermore, interactivity might suffer from interruptions in the internet connection leading to lag times (Wegge, 2006). In addition, conversations via webcam and microphone can also lead to a decrease in the transparency of the procedure. And finally, given the transmission of information via the internet, one is never completely immune to the possibility that the conversation is unknowingly recorded, which in turn can lead to fears of surveillance. Thus, based on earlier findings as well as on Gilliland’s fairness model, social presence theory, and Potosky’s framework, we predict:

- **Hypothesis 5a**. Interviewees perceive videoconference interviews as less fair than FTF interviews.

In addition to mean differences concerning fairness perceptions of different interviews, Basch et al. (2020) also found that ratings of expected social presence differed between the interview conditions and that these social presence ratings were related to the expectation to be able to use IM tactics. This in turn predicted fairness perceptions of different interview media. However, in that study, participants did not actually experience an interview. Nevertheless, we suppose that the assumed mediation path generalizes to actual interviews. Therefore, we predict:

- **Hypothesis 5b**. The relationship between interview medium and fairness perceptions is serially mediated by perceived social presence and IM.

Even though previous studies found that technology-mediated interviews are perceived more negatively than FTF interviews, these interviews also have obvious advantages such as the greater flexibility of videoconference interviews. Due to the independency of travel needs, dates for an interview can be arranged and coordinated more easily. This increased flexibility makes the coordination of the selection process easier both for applicants and for interviewers. Nevertheless, these advantages were often disregarded in previous studies. However, we assume that the lower perceptions of videoconference interviews should mainly be found for fairness variables whereas we assume that these interviews are perceived more positively than FTF interviews when perceived flexibility is taken into account as an additional applicant perception variable. Accordingly, we assume:

- **Hypothesis 6**. Interviewees perceive videoconference interviews as more flexible than FTF interviews.

Even though privacy concerns are not a common variable in applicant reaction research, they are relevant in the context of technology-mediated selection procedures. Privacy concerns are defined as the concern that the use of technology can invade privacy in general and personal data in particular. Accordingly, surveillance as one of Potosky’s (2008) media attributes also deals with privacy concerns (Malhotra, Kim, Agarwal, Tech, & Peachtree, 2004). We are not aware of any previous studies that considered privacy concerns as a dependent variable for the comparison between videoconference versus FTF interviews. However, Langer et al. (2017) found that asynchronous video interviews induced more privacy concerns compared to videoconference interviews. Although both types of interviews are conducted via the internet, a recording of the interview in asynchronous video interviews is obligatory whereas it is unusual for videoconference interviews. When comparing videoconference interviews and FTF interviews, however, videoconference interviews are probably more likely to induce privacy concerns because they are conducted via the internet and almost every information in the internet is vulnerable to third party access. Therefore, we assume:

- **Hypothesis 7**. Videoconference interviews will lead to more privacy concerns than FTF interviews.

## Method

Given that we predicted differences between videoconference versus FTF interviews, we could not test our hypotheses in a high-stakes selection setting. Therefore, we tested them in simulated selection interviews. This allowed us to collect all variables of interest in a setting that comes at least relatively close to actual selection interviews. Furthermore, previous research also revealed that interview performance ratings from interview simulations can be criterion valid for the prediction of performance in job simulations (Oostrom, Melchers, Ingold, & Kleinmann, 2016), of academic performance (Day & Carroll, 2003; Klehe & Latham, 2006), and of actual job performance (Ingold, Kleinmann, König, Melchers, & Van Iddekinge, 2015).

### Sample

A total of 142 student participants completed a pre-interview questionnaire (see below). However, only 114 of these participants subsequently took part in an actual interview (57 in each of two interview conditions). One out of these 114 participants did not complete the post-interview questionnaire. Furthermore, due to data transmission problems during the pre- or post-interview questionnaire, there were missing data for another pre-interview questionnaire and two additional post-interview questionnaires.^1^ Given that the main focus of our study was on performance differences between different interview media and on possible reasons for these differences, we could not use data from participants who only completed the pre-interview questionnaire. However, for all the analyses for which this was possible, we decided to use data from the four participants with missing data.

The 114 participants in our final sample (73% females; age: *M* = 23.96 years) came from different courses of study (41% Bachelor, 39% Master, 18% PhD) at a German university. The majority of them were holding a job and on average their weekly working time was 9.81 h (*SD* = 12.84). Participants had a mean of 3.67 (*SD* = 3.84) previous interviews. Additionally, 21% of the participants already had experience with technology-mediated interviews.

### Procedure

As noted above, the study had three parts. For the first part, participants were sent an online questionnaire in which they had to complete demographic questions and questions concerning their experience with selection interviews in general and technology-mediated interviews in particular. Eventually, participants were randomly assigned either to the FTF or to the videoconference interview condition. The respective interview was described in detail and then participants had to answer questions concerning perceptions of the respective interview. At the end of this questionnaire, participants were asked to sign up for an interview during the subsequent two to three weeks.

The second part of the study consisted of the interview itself, which contained a set of questions that were suitable for university graduates. The interviews were conducted by one of four different interviewers. Furthermore, FTF interviews were videotaped by camera and videoconference interviews were recorded via screencasts. Each interviewee was once rated directly after the interview (live rating) and once by another member of the interviewer team based on the video recordings (recorded rating).

After the participants had completed the interview, they were sent the final online questionnaire. It contained items concerning fairness perceptions, perceived quality of eye contact, perceived social presence, IM during the interview, perceived flexibility, and privacy concerns.

#### Interview Conditions

The interviews were conducted either face-to-face or via videoconference. In both conditions, participants were told to dress adequately as if they were to go through an actual selection interview. The FTF interview was always conducted in the same room at the university and the interviewer for the videoconference interviews was always using this room as well. For videoconference interviews, we used the free version of Skype (www.skype.com). Additionally, participants in the videoconference condition were told to complete the interview in a quiet environment with a stable internet connection.

### Measures

Unless indicated otherwise, all items were rated on 5-point rating scales ranging from 1 = *strongly disagree* to 5 = *strongly agree*. Measures for which no German version was available were translated to German for the present study and were checked by backtranslation. All items can also be found in Table A1 in the Appendix.

#### Structured Interview

The interview consisted of 13 questions (six past behavioral questions and seven future-oriented questions) that covered the three dimensions Perseverance (e.g., “You probably know the following situation from your studies. You attended a course that did not meet your expectations, but was part of the compulsory curriculum. How did you deal with this?”), Organizing behaviors (e.g., “Remember another situation from your studies. You had to familiarize yourself with a completely new topic, for example for a lecture or a seminar paper at the university. Describe briefly how you proceeded in this or a similar situation”), and Assertiveness (e.g., “Please imagine the following situation. You are the coordinator of a project. Recently it has become increasingly common for your colleagues not to meet the deadlines you have set. This has already led to you being increasingly called to account by your supervisor. Today, it has happened again that two of your colleagues have not submitted their documents as agreed. What would you do in this situation?”). The questions were taken from another study that had developed interview questions from a large pool of critical incidents (Ingold et al., 2015). The questions were slightly modified to be suitable for the present study. Furthermore, in line with best-practice recommendations (Campion, Palmer, & Campion, 1997), we used a highly structured interview so that interviewers were not allowed to probe responses for any of the different interview questions.

The interviewer team consisted of two work and organizational psychology PhD students and two work and organizational psychology master’s students. The master’s students were already experienced in conducting interviews from previous student jobs in the department of Work and Organizational Psychology and from previous HR-related jobs. Furthermore, all interviewers and raters received several hours of frame-of-reference training (Melchers, Lienhardt, von Aarburg, & Kleinmann, 2011; Roch, Woehr, Mishra, & Kieszczynska, 2012). In the training, all interviewers were introduced to basics of the rating processes, the interview, and definitions and examples of poor, average, and excellent answers. Interviewers practiced the rating process, worked with the scoring instructions, discussed their ratings, and received feedback on their ratings.

The data collection was carried out during two different periods of time with one interview team for each period. For each interview, one interviewer was randomly assigned to conduct the live interview. The other interviewer then automatically evaluated the recorded interview.

For each question, ratings of interviewees’ performance were made on 5-point scales ranging from 1 = *poor* to 3 = *average* to 5 = *excellent* for which descriptive anchors were provided for poor, average, and excellent answers. When ratings for a question differed two or more points on the 5-point scale, the interviewer and the second rater discussed their observations and ratings. Although interviewers did not have to agree with each other, most differences could be resolved after a short discussion, meaning that the ratings did not usually differ by more than one point. In view of the findings by Van Iddekinge et al. (2006), who found a difference between live ratings versus ratings based on video recordings of the same interviews, we decided to treat the live rating and the recorded rating as two different ratings and not to combine them to a mean value.

To determine the interrater reliability, we used one-way random ICCs. Before the discussion of the ratings, the interrater reliability (ICC 1.1, Shrout & Fleiss, 1979) for the average rating across all 13 questions was 0.90 and after the discussion, it was 0.93.^2^ We used the ICC 1.1 (one-way random, single-measure) because not every case was rated by every rater, the raters were randomly assigned to the interviewees, and the ratings were not combined to a mean of the different raters. Thus, the ICCs represent the reliability of the overall average across all 13 questions of a single interviewer. Given the slightly improved interrater reliability, we used the values after the discussion.

#### Impression Management

In the post-interview questionnaire, participants had to rate their usage of IM with four items from Tsai, Chen, and Chiu (2005) (e.g., “In this interview, I was able to describe my skills and abilities in an attractive way,” α = 0.70). Regarding the moderate internal consistencies and since Tsai et al. had taken the items from different scales, we additionally conducted a confirmatory factor analysis (CFA). The resulting fit of a single-factor model was very good (CFI = 1.00, TFI = 0.99, RMSEA = 0.04).

#### Perceived Quality of Eye Contact

Perceived quality of eye contact was measured with two self-developed items for the interviewee and two self-developed items for the interviewer. Participants answered these items (e.g., “In this interview, I found it easy to keep eye contact with the interviewer” and “In this interview, I often sought eye contact with the interviewer”) in the post-interview questionnaire (α = 0.68). Interviewers also answered to their items after the interview (“I found it easy to keep eye contact with the interviewee” and “The interviewee kept eye contact in a comfortable way”, α = 0.80).

#### Social Presence

Four items developed and validated by Short et al. (1976) were used to measure social presence. Participants were asked to rate their perception of the conversation situation in the respective interview (“How did you perceive the conducted interview?”) in the post-interview questionnaire on 5-point bipolar adjective scales on the following adjective pairs: *insensitive–sensitive*, *cold–warm*, *active–passive,* and *impersonal–personal* (α = 0.76). These items have proven to be suitable to measure presence in different studies (e.g., Tang, Wang, & Norman, 2013).

#### Fairness Perceptions

We used four subscales from the Selection Procedural Justice Scale (Bauer et al., 2001) in a German translation from Manzey and Gurk (2005) to measure participants’ fairness perceptions. Participants had to rate the items once in the pre-interview questionnaire and once in the post-interview questionnaire. These four subscales represented predictive job-relatedness (e.g., “Doing well in such an interview means I could do well on the job”, α_pre_ = 0.72, α_post_ = 0.76), chance to perform (e.g., “In such an interview I can really show my skills and abilities”, α_pre_ = 0.83, α_post_ = 0.84), two-way communication (e.g., “There is enough communication during such an interview”, α_pre_ = 0.72, α_post_ = 0.72), and global fairness (e.g., “I think that such an interview is a fair way to select people”, α_pre_ = 0.72, α_post_ = 0.76).

#### Perceived Flexibility

To measure perceived flexibility, we used three items from a previous study (Basch & Melchers, 2019) (e.g., “Such an interview offers great temporal and geographical flexibility to applicants”, α_pre_ = 0.81, α_post_ = 0.72).

#### Privacy Concerns

Privacy concerns were measured with five items (e.g., “In such an interview I am worried about my privacy”, α = 0.74) used in a study by Langer et al. (2017). Two of these items were initially taken from Malhotra et al. (2004), one from Smith, Milberg, and Burke (1996), and two were developed by Langer et al. (2017).

## Results

### Preliminary Analyses

Preliminary analyses by means of *t* tests revealed that the two experimental groups did not differ concerning sex, age, mother tongue, working hours per week, or prior interview experience. Additionally, groups were comparable with respect to their high school grade point average (all *t*s < 1.05, all *p*s > 0.30). The means, standard deviations, and intercorrelations for all study variables can be seen in Table 1.

**Table 1:** Descriptive information and correlations for all study variables

Variable	*M*	*SD*	1	2	3	4	5	6	7	8	9	10	11	12	13	14
1. Age	23.96	2.77														
2. Sex	0.27	0.45	0.10													
3. Interview condition	0.50	0.50	0.10	0.10												
4. Interview performance live rating	3.87	0.38	− 0.09	− 0.10	− 0.23*											
5. Interview performance recorded rating	3.81	0.38	− 0.05	− 0.10	− 0.14	0.85**										
6. Impression management	3.03	0.57	− 0.13	0.07	− 0.23*	0.29**	0.25*	*0.70*								
7. Eye contact (self)	3.46	0.97	− 0.07	0.02	− 0.53**	0.19*	0.15	0.47**	*0.68*							
8. Eye contact (interviewer)	3.50	0.82	0.00	− 0.05	− 0.31**	0.24*	0.19*	0.27**	0.26**	*0.80*						
9. Social presence	3.37	0.70	− 0.13	0.10	− 0.21*	0.04	0.01	0.58**	0.39**	0.17	*0.76*					
10. Fairness pre	3.23	0.53	0.00	0.04	− 0.21*	0.08	0.18	0.13	0.11	0.16	0.18	*0.86*				
11. Flexibility pre	3.52	0.79	0.00	0.03	0.63**	− 0.13	− 0.13	− 0.11	− 0.28**	− 0.27**	− 0.02	− 0.04	*0.81*			
12. Fairness post	3.31	0.53	− 0.14	0.10	− 0.11	0.17	0.15	0.63**	0.36**	0.16	0.66**	0.32**	0.02	*0.86*		
13. Flexibility post	4.10	0.79	− 0.04	0.14	0.60**	− 0.14	− 0.03	− 0.02	− 0.23*	− 0.14	0.01	− 0.12	0.65**	0.04	*0.72*	
14. Privacy concerns	2.70	0.61	0.06	0.03	0.40**	− 0.12	− 0.15	− 0.11	− 0.25**	− 0.15	− 0.16	− 0.17	0.23*	− 0.11	− 0.23*	*0.74*

*N* = 111–114. Sex was coded 0 = female, 1 = male. Interview condition was coded 0 = face-to-face, 1 = videoconference. Fairness represents the mean of the four fairness subscales. Values in the diagonal represent coefficient alphas

*p < 0.05; **p < 0.01

### Performance Differences in FTF versus Videoconference Interviews

Hypothesis 1 stated that there would be a difference between interview performance ratings in FTF interviews and videoconference interviews. As can be seen in Table 2, ratings were indeed higher in the FTF condition than in the videoconference condition. To formally test these differences, we conducted a multivariate analysis of variance (MANOVA) with the interview conditions as the independent variable and the live ratings and recorded ratings as the dependent variables. As mentioned above, we decided to treat ratings based on live interviews and ratings based on recorded interviews as separate variables because of the results by Van Iddekinge et al. (2006) who found significant differences between ratings in FTF interviews versus ratings that were based on video recordings of the same interviews. Accordingly, we included the different ratings as separate dependent variables in a MANOVA.

**Table 2:** Means, standard deviations, effect sizes, and *p* values for the comparison of performance-related dependent variables between FTF and videoconference interviews

Dependent variable	Face-to-face		Videoconference		Cohen’s *d*	*p* value
	*M*	(*SD*)	*M*	(*SD*)		
Interview ratings						
Live rating	3.96	(0.39)	3.78	(0.36)	0.43	0.007
Recorded rating	3.86	(0.41)	3.75	(0.34)	0.27	0.06
Performance-related variables						
Impression management	3.47	(0.55)	3.18	(0.66)	0.48	0.007
Perceived quality of eye contact self-reported	3.97	(0.75)	2.96	(0.91)	1.21	< 0.001
Perceived quality of eye contact interviewer	3.75	(0.82)	3.25	(0.74)	0.64	< 0.001
Social presence	3.51	(0.68)	3.22	(0.70)	0.42	0.01

The *p* values represent the results of one-tailed *t* tests

In line with Hypothesis 1, the MANOVA revealed a significant difference, Wilk’s λ = 0.92, *F*(2, 110) = 4.60, *p* = 0.01. Separate one-tailed *t* tests revealed that this effect was mainly driven by the difference for the live ratings, *t*(111) = 2.50, *p* = 0.007, *d* = 0.43. In contrast to this, the difference for the ratings on the basis of the recorded interviews just failed significance, *t*(111) = 1.51, *p* = 0.06, *d* = 0.27. A significant effect was also found when we used the mean of live and recorded rating, *t*(111) = 1.99, *p* = 0.02, *d* = 0.39.

To formally evaluate differences between live and recorded ratings, we compared the live ratings versus the ratings by means of a 2 × 2 ANOVA (Condition × Rating). This ANOVA revealed a significant effect for the interaction term, *F*(1, 111) = 4.26, *p* = 0.04. In line with Van Iddekinge et al. (2006), subsequent paired-samples *t* test revealed a significant difference between the two ratings in the FTF condition, *t*(56) = 3.02, *p* = 0.004, *d* = 0.40. In contrast, no such effect was found for the videoconference interviews, *t*(56) = 0.75, *p* = 0.46, *d* = 0.06.

Hypothesis 2a predicted that IM mediates the relationship between the interview condition and interview performance ratings, so that less IM in videoconference interviews leads to the lower performance in these interviews. To test differences in IM between the conditions, we first conducted a *t* test that revealed a significant difference, *t*(110) = 2.49, *p* = 0.007, *d* = 0.48, which reflects lower IM usage in the videoconference interview.

**Fig. 1:**
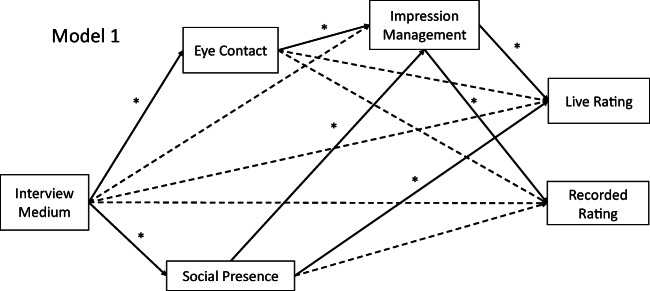
Structural equation model for the mediation analyses related to Hypotheses 2a, 2c, 3b, and 4. Non-significant paths are shown with dashed lines

To test the predicted mediation effect from Hypothesis 2a (as well as the subsequent mediation hypotheses), we conducted mediation analyses using structural equation modeling and tested the significance of the indirect paths using the PROCESS macro from Hayes (2018). The weights from the structural equation models are shown in Table 3 and results for the indirect effects are shown in Table 4 (cf. Fig. 1 for a graphical summary). In line with Hypothesis 2a, significant mediation effects were found for both ratings of interviewees’ performance.

**Table 3:** Regression weights for the structural equation model predicting interview performance ratings according to the model in Fig. 1

Dependent variables and predictors	*B*	*SE*	*p* value
Eye contact			
Interview condition	− 1.03	0.16	< 0.001
Social presence			
Interview condition	− 0.30	0.13	0.02
Impression management			
Interview condition	0.02	0.11	0.89
Eye contact	0.18	0.06	0.001
Social presence	0.42	0.07	< 0.001
Interview performance live rating			
Interview condition	− 0.14	0.08	0.08
Impression management	0.22	0.07	0.002
Social presence	− 0.12	0.06	0.04
Eye contact	0.01	0.04	0.91
Interview performance recorded rating			
Interview condition	− 0.06	0.08	0.49
Impression management	0.21	0.07	0.004
Social presence	− 0.12	0.06	0.05
Eye contact	0.01	0.04	0.77

*N* = 111. Interview condition was coded 0 = face-to-face, 1 = videoconference

**Table 4:** Results for the indirect paths of the different mediation analyses concerning Hypotheses 2a, 2c, 3b, and 4

Model	*IE*_med_	*SE*_Boot_	95% CI
Interview condition → IM → live rating	− 0.04	0.02	[− 0.10, − 0.01]
Interview condition → IM → recorded rating	− 0.04	0.02	[− 0.10, − 0.01]
Interview condition → self-report eye contact → IM → live rating	− 0.04	0.02	[− 0.11, − 0.02]
Interview condition → self-report eye contact → IM → recorded rating	− 0.04	0.02	[− 0.10, − 0.01]
Interview condition → eye contact (interviewer) → IM → live rating	− 0.01	0.01	[− 0.03, 0.00]
Interview condition → eye contact (interviewer) → IM → recorded rating	− 0.01	0.01	[− 0.03, 0.00]
Interview condition → social presence → live rating	0.00	0.02	[− 0.03, 0.03]
Interview condition → social presence → recorded rating	0.00	0.02	[− 0.03, 0.04]
Interview condition → social presence → IM → live rating	− 0.03	0.01	[− 0.07, − 0.01]
Interview condition → social presence → IM → recorded rating	− 0.02	0.01	[− 0.06, − 0.01]
Interview condition → social presence → IM → fairness	− 0.05	0.02	[− 0.11, − 0.01]

*N* = 111. The 95% confidence interval for the effects was obtained by the bias-corrected bootstrap with 10,000 resamples. *IM* = impression management. *IE*_*med*_ = completely standardized indirect effect of the mediation. *SE*_*Boot*_ = standard error of the bootstrapped effect sizes

Hypothesis 2b predicted that participants would perceive a higher quality of eye contact in FTF interviews compared to videoconference interviews. A MANOVA with interviewees’ self-reported post-interview ratings as well as with the interviewer ratings of perceived eye contact revealed a significant difference, Wilk’s λ = 0.69, *F*(2, 109) = 24.12, *p* < 0.001. Separate follow-up ANOVAs confirmed significant differences for the self-report measure, *F*(1, 110) = 41.80, *p* < 0.001, *d* = 1.21, as well as for the ratings by the interviewer, *F*(1, 112) = 11.71, *p* = 0.001, *d* = 0.64 (cf. Table 2). Hypothesis 2b was therefore supported.

In Hypothesis 2c, we predicted that the relationship between the interview condition and interview performance ratings would be serially mediated by perceived quality of eye contact and IM. To examine this, we conducted analyses for interviewees’ ratings of IM and of perceived eye contact and also for interviewees’ ratings of IM and interviewer-rated quality of eye contact (Table 3). The indirect effect was significant both for the paths via self-reported quality of eye contact and interviewer-reported quality of eye contact (Table 4). Hypothesis 2c was therefore supported.

Hypothesis 3a stated that perceived social presence would be higher in FTF interviews compared to videoconference interviews. In line with this prediction, the corresponding means in Table 2 were higher in the FTF condition and a *t* test revealed a significant difference, *t*(110) = 2.22, *p* = 0.01, *d* = 0.42. Hypothesis 3a was therefore supported.

To evaluate Hypothesis 3b that predicted a mediation effect of interview condition on interview performance ratings via perceived social presence, we again conducted a mediation analysis. In contrast to Hypothesis 3b, the results were neither significant for the live nor for the recorded ratings (Table 4).

Hypothesis 4 predicted a serial mediation of the relationship between interview condition on interview performance ratings via perceived social presence and IM. In line with Hypothesis 4, the serial mediation path turned out to be significant for both the live and the recorded ratings (Table 4).^3^

### Interviewees’ Perceptions of the Different Interviews

Hypothesis 5a stated that videoconference interviews are perceived as less fair in comparison to FTF interviews. The means, standard deviations, and effect sizes for the four fairness subscales can be seen in Table 5. To evaluate this, we used a 2 × 2 MANOVA (Condition × Pre/Post) with all the fairness subscales. In contrast to our expectation, the MANOVA for the main effect of condition, Wilk’s λ = 0.95, *F*(4, 106) = 1.39, *p* = 0.24, and the Condition × Pre/Post interaction, Wilk’s λ = 0.96, *F*(4, 106) = 1.06, *p* = 0.38, failed to reach significance. Hypothesis 5a was therefore not supported. However, a *t* test with the aggregate score of the pre-questionnaire turned out to be significant, *t*(111) = 2.28, *p* = 0.03.

**Table 5:** Means, standard deviations, effect sizes, and *p*-values for the comparisons of interviewee perceptions variables between FTF and videoconference interviews

Dependent variable	Face-to-face		Videoconference		Cohen’s *d*	*p* value
	*M*	(*SD*)	*M*	(*SD*)		
Pre-interview						
Predictive job-relatedness	3.42	(0.75)	3.27	(0.76)	0.20	0.14
Opportunity to perform	3.01	(0.69)	2.80	(0.62)	0.32	0.05
Two-way communication	3.57	(0.61)	3.25	(0.68)	0.50	0.004
Global fairness	3.46	(0.68)	3.34	(0.79)	0.16	0.14
Fairness (aggregate score)	3.34	(0.51)	3.11	(0.53)	0.44	0.01
Flexibility	3.04	(0.65)	4.03	(0.59)	− 1.59	< 0.001
Post-interview						
Predictive job-relatedness	3.06	(0.80)	2.94	(0.78)	0.15	0.20
Opportunity to perform	2.94	(0.66)	2.78	(0.68)	0.24	0.10
Two-way communication	3.89	(0.69)	3.84	(0.63)	0.08	0.36
Global fairness	3.48	(0.63)	3.32	(0.82)	0.22	0.14
Fairness (aggregate score)	3.36	(0.51)	3.25	(0.55)	0.21	0.13
Flexibility	3.63	(0.59)	4.38	(0.55)	− 1.31	< 0.001
Privacy concerns	2.46	(0.46)	2.94	(0.65)	− 0.85	< 0.001

The *p* values represent the results of one-tailed *t* tests

**Fig. 2:**
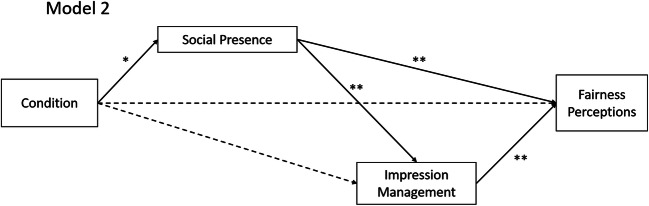
Structural equation model for the mediation analyses related to Hypothesis 5b. Non-significant paths are shown with dashed lines

Hypothesis 5b predicted that the effect of the interview condition on fairness perceptions is serially mediated via perceived social presence and IM. We used the mean across all fairness scales for the post-interview ratings. In line with our hypothesis, the indirect effect turned out to be significant (cf. Tables 4 and 6 and Fig. 2).

**Table 6:** Regression weights for the structural equation model predicting fairness perceptions

Dependent variables and predictors	β	*SE*	*p* value
Social presence			
Interview condition	− 0.29	0.13	0.03
Impression management			
Interview condition	− 0.14	0.10	0.14
Social presence	0.49	0.07	< 0.001
Fairness perceptions			
Interview condition	0.08	0.07	0.29
Social presence	0.34	0.06	< 0.001
Impression management	0.32	0.07	< 0.001

*N* = 111. Interview condition was coded 0 = face-to-face, 1 = videoconference

Hypothesis 6 predicted that there would be a difference of perceived flexibility in favor of videoconference interviews. In line with this, we found significant differences in favor of videoconference interviews both before the interview, *t*(111) = − 8.61, *p* < 0.001, *d* = –1.59, and after it, *t*(110) = − 6.93, *p* < 0.001, *d* = –1.31.

Finally, Hypothesis 7 predicted that videoconference interviews induce more privacy concerns than FTF interview. In line with this hypothesis, a *t* test found a significant difference, *t*(110) = − 4.54, *p* < 0.001, *d* = –0.85 (Table 5).

## Discussion

The purpose of this study was to investigate possible factors that contribute to differences concerning ratings of interviewees’ performance in FTF versus videoconference interviews as well as differences in their perceptions of these interviews. An overview of the results concerning the different hypotheses is provided in Table 7. In line with previous research, we found that interviewees received lower ratings in videoconference interviews compared to FTF interviews. Our results suggest that the reasons for these differences can be found on the side of the interviewer as well as on the side of the interviewee. Furthermore, we found that interviewees reported more privacy concerns regarding videoconference interviews in comparison to FTF interviews but perceived them as more flexible.

**Table 7:** Summary of the results

Hypotheses	Confirmed?
Hypothesis 1: Interviewees receive lower performance ratings in videoconference interviews compared to FTF interviews.	Yes
Hypothesis 2a: Interviewees use lower levels of IM in videoconference interviews than in FTF interviews which in turn leads to the lower performance in videoconference interviews.	Yes
Hypothesis 2b: Interviewees will perceive a lower quality of eye contact in videoconference interviews compared to FTF interviews.	Yes
Hypothesis 2c: The relationship between interview medium and interview performance ratings is serially mediated by perceived quality of eye contact and IM.	Yes
Hypothesis 3a: Perceived social presence is higher in FTF interviews compared to videoconference interviews.	Yes
Hypothesis 3b: The relationship between interview medium and interview performance rating is mediated by social presence.	No
Hypothesis 4: The relationship between interview medium and interview performance ratings is serially mediated by social presence and IM.	Yes
Hypothesis 5a: Interviewees perceive videoconference interviews as less fair than FTF interviews	No
Hypothesis 5b: The relationship between interview medium and fairness perceptions is serially mediated by social presence and IM.	Yes
Hypothesis 6: Interviewees perceive videoconference interviews as more flexible than FTF interviews.	Yes
Hypothesis 7: Videoconference interviews will lead to more privacy concerns than FTF interviews.	Yes

The differences between interviewees’ performance ratings in FTF versus videoconference interviews fit in with previous studies that already found lower performance ratings in technology-mediated interviews compared to FTF interviews (Blacksmith et al., 2016; Melchers et al., 2016; Sears et al., 2013). However, the reasons for these differences remained largely unclear in previous studies. Thus, the present study contributes to the literature by providing insights into the factors that contribute to these differences.

With respect to the side of the interviewer, we found that interviewees in FTF interviews were rated lower when the ratings were based on video recordings. A similar effect was found by Van Iddekinge et al. (2006), who compared FTF and videotaped ratings of the same interview. In contrast to this, no significant difference was found between live versus recorded interview ratings in the videoconference condition, which replicates a similar finding by Langer et al. (2017). Thus, it seems unlikely that factors such as differences in raters’ cognitive load contributed to differences in their ratings in the FTF interview. However, the finding that interviewer-rated perceived quality of eye contact was a mediator of the effect of interview condition on interview performance ratings stresses the role of non-verbal cues on interviewer evaluations (cf. Burnett & Motowidlo, 1998) and these non-verbal cues are impaired in technology-mediated communication given their lower social bandwidth. Furthermore, another factor might be perceived social presence on the side of the interviewer. Therefore, it might also be that the impairment of social presence can be an influencing factor leading to less mutual sympathy, which eventually impairs interviewer ratings.

Concerning the side of the interviewee, our study is also one of the first studies that shed light on possible mediators of performance differences in FTF and videoconference interviews. One of these mediators turned out to be IM. Participants perceived that it was more difficult to demonstrate IM tactics in videoconference interviews. Possible reasons for such impairments include time delays (Blacksmith et al., 2016), impaired feedback from the interviewer (Fullwood & Finn, 2010), the presence of one’s own picture during the videoconference communication (Horn & Behrend, 2017), and lower social bandwidth in videoconference interviews (Potosky, 2008). Furthermore, in line with previous studies (Barrick et al., 2009), the present study found that IM usage had a significant correlation with interview ratings and mediation analyses supported IM usage as a mediator of the effects of interview condition on interview performance.

The quality of perceived eye contact turned to be another antecedent of rating differences. Specifically, we found lower values for self-reported and for interviewer-rated quality of eye contact in videoconference interviews as well as a serial mediation effect of the interview medium on self-reported quality of eye contact and IM on interview performance ratings. Making eye contact is impeded in videoconference interviews as it can only be established by looking into the camera (Bohannon et al., 2013), which makes it even more difficult to keep track of the impressions of the conversation partner and therefore also with non-verbal IM tactics.

Additionally, significant serial mediation effects were found when we considered perceived social presence or the perceived quality of eye contact. Interviewees not only rated perceived social presence lower in videoconference interviews, but social presence also had an impact on IM behavior and ultimately on performance ratings. The difference in perceived social presence between videoconference and FTF interviews is in line with social presence theory (Short et al., 1976). Thus, despite the technological advances and the relatively comprehensive transfer of the different social cues, social presence still lags behind due to technological mediation. In contrast, according to media richness theory (Daft & Lengel, 1986), there should have been little difference between the media. Thus, based on the results, it seems more plausible to assume that social presence is more a facet of human perception than an attribute of a medium.

In addition to insights into the factors that contribute to performance differences between FTF versus videoconference interviews, our study also leads to a better understanding of interviewees’ perceptions of these interviews. Specifically, we could not find significant differences for most of the fairness perceptions of these interviews. Instead, there was only a slight difference for the aggregate score of fairness subscales in the pre-interview questionnaire but this difference was no longer significant after the interview. Thus, on the one hand, fairness perceptions concerning FTF and videoconference interviews were relatively similar so that reports of larger differences from older studies (Blacksmith et al., 2016) or findings that were only based on hypothetical interviews (Basch et al., 2020) might not generalize to actual interviewees in current selection processes. On the other hand, even though fairness perceptions after the interview did not differ significantly, mediation analyses nevertheless found that the interview medium affected these perceptions indirectly. Specifically, in line with results from Basch et al. (2020), differences in fairness perceptions between FTF and videoconference interviews were serially mediated by perceived social presence and IM. Thus, technology mediation and, therefore, the missing physical appearance of one’s conversation partner can be seen as a barrier for potential applicants to present themselves in a positive light, which leads to less favorable perceptions of videoconference interviews.

Finally, we also considered two applicant perception variables beyond fairness. One of these variables was perceived flexibility. According to our results, interviewees perceived videoconference interviews as more flexible than FTF interviews. Thus, this advantage of technology-mediated interviews is well recognized by applicants. The other perception variable was privacy concerns, which was rated higher in videoconference interviews compared to FTF interviews. Although both interviews were videotaped in our study, the transmission of information via the internet seems to be a factor of which many people are both aware and frightened of (Malhotra et al., 2004).

### Limitations and Lines for Future Research

Even though the present study provides several important insights into differences between FTF versus videoconference interviews, it is not without limitations. The first of these limitations concerns the student sample. Students may be more familiar with modern communication technologies than the general population. This could lead to an underestimation of the difference between the interview conditions compared to the general population. Additionally, due to the simulated selection setting, participants probably did not complete the interview with the same motivation as applicants in a high-stakes selection setting. However, given the expected differences concerning interviewees’ performance ratings, a comparable study could not be conducted in a field setting because of ethical reasons. However, results from quasi-experimental field studies covered in the meta-analysis by Blacksmith et al. (2016) also found higher interview ratings in FTF interviews than in technology-mediated interviews.

Another limitation of the present study is that it is unclear to which degree our results generalize to less structured interviews. Our interview was highly structured regarding the administration and evaluation of the interview. Even though structure is beneficial for the psychometric properties of interviews, most interviews in field settings use less structure (Chapman & Zweig, 2005; Roulin, Bourdage, & Wingate, 2019). Therefore, future research is necessary to evaluate the effects of technology mediation in less structured interviews.

A third limitation concerns our measure for perceived quality of eye contact. The internal consistency in the post-interview items only reached a moderate level and this was mainly because of the videoconference condition. In retrospect, it seems that the second item (“In this interview, I often sought eye contact with the interviewer”) was suboptimal given that keeping eye contact is difficult in the context of videoconference interviews because interviewees cannot fix the interviewer’s eyes and look into the camera at the same time in these interviews. This might have led to more attempts to *seek* eye contact because of the problems to *hold* good eye contact. Future research that wants to investigate the perceived quality of eye contact should take this into account.

A final limitation concerns the question, whether differences in interview ratings are related to differences concerning criterion-related validity. Given that social aspects such as perceived social presence and eye contact contributed to differences in interview ratings and also given findings by Burnett and Motowidlo (1998) that eye contact contributed to criterion-related validity of a structured interview beyond the content of interviewees’ answers, it might well be that there are differences in criterion-related validity of FTF versus videoconference interviews. Therefore, whether such differences can indeed be found should be investigated by further research.

### Practical Implications

Based on the findings concerning lower performance ratings in videoconference interviews, we recommend avoiding a mix of FTF and videoconference interviews in the same selection stage to give all applicants an equal chance to show their qualities. Although FTF and videoconference conversations have become more and more aligned over the years due to technological progress, organizations should not overlook that the interview medium affects applicants’ chances in a selection process.

Nevertheless, the use of videoconference interviews should not be abandoned, as it can lead to a reduction of costs in the recruiting process and to a larger applicant pool (Chapman & Webster, 2003). Furthermore, the experience of the coronavirus and the lockdowns that were introduced in many countries also stress the advantages of being able to use technology-mediated selection interviews. Nevertheless, attempts should be made to adapt videoconference communication in a way to reduce differences between FTF and videoconference interviews. Accordingly, high-resolution videoconference systems that allow as much non-verbal communication as possible should be used (Fullwood, 2007). In addition, it might be beneficial to implement and use newer systems to improve eye contact. These might implement a rotation of the image of the conversation partner around the horizontal axis that has shown to improve the perceived quality of eye contact (Jaklič, Solina, & Šajn, 2017) or a fixation of the camera behind the face of the communication partner that is projected on a transparent screen (Bondareva, Meesters, & Bouwhuis, 2006).

Even though there are several practical advantages of videoconference interviews, applicants should be aware of the potential limitations of videoconference interviews. Thus, it seems advisable for applicants to choose a FTF interview if they have the choice between the two interview media because this improves their chances for a job offer. However, if applicants have to go through a videoconference interview, they can try to establish eye contact by not looking at their screen but at the camera to give the interviewer a sense of eye contact. Fullwood and Doherty-Sneddon (2006) have already shown that eye contact can improve the memory performance of conversation partners in videoconference communication. In an interview context, this could also have positive effects on performance evaluations by interviewers.

Furthermore, given the obvious practical advantages of videoconference interviews such as their higher flexibility, it might be helpful to stress these advantages to applicants. Specifically, meta-analytic evidence by Truxillo, Bodner, Bertolino, Bauer, and Yonce (2009) found that giving an explanation concerning the advantages of selection instruments can improve applicant reactions on these instruments. Thus, explanations might also be a viable option to improve applicant perceptions of these interviews and their reactions to the selection process. In line with this possibility, a recent study by Basch and Melchers (2019) indeed found that an explanation related to the larger flexibility of technology-mediated interviews can have positive effects of the perceived flexibility and ultimately on organizational attractiveness. Thus, it would be conceivable to emphasize advantages of videoconference interviews prior to the selection process in order to prevent low fairness perceptions and poor applicant reactions such as lower organizational attractiveness (Hausknecht et al., 2004).

## Appendix

**Table A1:** Items used to measure the different variables. For all items, German translations were used in our study

Items used for the current study		Source
Pre-interview questionnaire		
Fairness perceptions	Job-relatedness: Doing well on such an interview means I could do well on the job I have in mind. A person who scored well on this interview will do well on the job I have in mind. Chance to perform: I could really show my skills and abilities in such an interview. Such an interview would allow me to show what my job skills are. Such an interview gives applicants the opportunity to show what they can really do. I would be able to show what I can in such an interview, Two-way communication: There would be enough communication in such an interview. I would be satisfied with the communication that occurred during such an interview. I would feel comfortable asking questions about such an interview if I had any. I would be comfortable with the idea of expressing my concerns in such an interview. Global fairness: I think that such an interview is a fair way to select people for the job I have in mind. I think that such interview itself would be fair.	Bauer et al. (2001) German translation from Manzey and Gurk (2005)
Perceived flexibility	Finding a suitable appointment for the interview would be easy with this method of interviewing. The entire process of such an interview would be very easy. Such an interview offers great temporal and geographical flexibility to applicants.	Basch and Melchers (2019)
Post-interview questionnaire		
Impression management	During the interview, I was able to describe my skills and abilities in an attractive way. During the interview, I was able to demonstrated my knowledge and expertise. During the interview, I was able to emphasize the qualities that I possess. During the interview, I was able to use friendly nonverbal cues like smiling and nodding.	Tsai et al. (2005)
Perceived quality of eye contact—interviewee	In this interview, I found it easy to keep eye contact with the interviewer. In this interview, I often sought eye contact with the interviewer.	Self-developed
Perceived quality of eye contact—interviewer	I found it easy to keep eye contact with the interviewee. The interviewee kept eye contact in a comfortable way.	Self-developed
Social presence	Impersonal—personal Insensitive—sensitive Cold—warm Passive—active	Short et al. (1976)
Fairness perceptions	Job-relatedness: Doing well on in this interview means I can do well on the job I have in mind. A person who scored well on this interview will do well on the job I have in mind. Chance to perform: I could really show my skills and abilities in this interview. This interview allowed me to show what my job skills are. This interview gives applicants the opportunity to show what they can really do. I was be able to show what I can in this interview. Two-way communication: There was enough communication in this interview. I am satisfied with the communication that occurred during the interview. I felt comfortable asking questions about the interview if I had any. I was comfortable with the idea of expressing my concerns in the interview. Global fairness: I think that this interview is a fair way to select people for the job I have in mind. I think that the interview itself is fair.	Bauer et al. (2001) German translation from Manzey and Gurk (2005)
Perceived flexibility	Finding a suitable appointment for the interview was easy with this method of interviewing. The entire process of this interview was very easy. Such an interview offers great temporal and local flexibility to applicants.	Basch and Melchers (2019)
Privacy concerns	In such an interview, it is important to me to keep my privacy intact. In such an interview, I am concerned about my privacy.	Malhotra et al. (2004)
	Such interviews threaten applicants’ privacy. Private data submitted during such interviews could be misused.	Langer et al. (2017)
	During this interview, I provided data that will be stored safely (reverse scored)	Smith et al. (1996)
Example interview questions and descriptive anchors taken from Ingold et al. (2015)		
Interview question		Descriptive anchor
“You probably know the following situation from your studies. You attended a course that did not meet your expectations, but was part of the compulsory curriculum. How did you deal with this?” (Perseverance)		5) Regularly attends the course and also uses means to increase his/her motivation or uses means to change the situation. 3) Temporary absence from the course, but completion of the course. 1) Cancels the course or requires several attempts to complete it.
“Remember another situation from your studies. You had to familiarize yourself with a completely new topic, for example for a lecture or a seminar paper at the university. Describe briefly how you proceeded in this or a similar situation.” (Organizing behaviors)		5) Describes a structured approach: getting an overview, researching using various sources (internet, library, experts), skimming the literature, structuring the subject, setting priorities, etc. 3) Describes an approach that is only slightly planned/structured, e.g. uses only one source of information. 1) Does not make a plan, just reads into topics or does nothing
“Please imagine the following situation. You are the coordinator of a project. Recently it has become increasingly common for your colleagues not to meet the deadlines you have set. This has already led to you being increasingly called to account by your supervisor. Today, it has happened again that two of your colleagues have not submitted their documents as agreed. What would you do in this situation?” (Assertiveness)		5) Confronts colleagues and makes clear their personal responsibility for the success of the project and the need to meet deadlines. He/she makes it clear that he/she will not tolerate such behavior and, if necessary, asks for a discussion with the next higher-level supervisor, if no change should occur. Makes concrete suggestions for solutions. 3) Mentions in passing that he/she does not find it okay if the deadlines are not met. 1) Says nothing and hopes that the situation changes.

## Footnotes

1 The 28 participants who only completed the pre-interview questionnaire were younger than the remaining 114 participants, *t*(140) = 2.09, *p* = 0.04. None of the other demographic or dependent variables differed between these two groups, all *t*s < 1.44, all *p*s > .14. With regard to the missing post-interview questionnaires, no significances between any of the pre-interview questionnaire measures or the interview performance ratings were found, all *t*s < 1.77, all *p*s > 0.07.

2 Looking at the two groups separately, the ICCs were 0.90 in the FTF condition before the discussion and also after the discussion. For the videoconference condition, they were 0.86 before the discussion and 0.94 after it.

3 When we repeated all analyses separately for the past-behavior and future-oriented questions, the basic pattern of the results was largely comparable for both types of questions. Specifically, the results for Hypotheses 1, 2a, 3b, and 4 followed the same pattern except for single mediation analyses for the recorded ratings of the future-oriented questions (Hypotheses 2a and 4). Additionally, for Hypothesis 2c, all indirect effects via eye contact and impression management on live and recorded ratings failed to reach significance for the future-oriented questions.
